# Mechanism of Vascular Injury in Transcatheter Aortic Valve Replacement

**DOI:** 10.17691/stm2021.13.3.01

**Published:** 2021-06-28

**Authors:** E.A. Ovcharenko, K.U. Klyshnikov, A.A. Shilov, N.A. Kochergin, M.A. Rezvova, N.V. Belikov, V.I. Ganyukov

**Affiliations:** Head of Laboratory, Department of Experimental Medicine; Research Institute for Complex Issues of Cardiovascular Diseases, 6 Sosnovy Blvd, Kemerovo, 650002, Russia; Researcher, Department of Experimental Medicine; Research Institute for Complex Issues of Cardiovascular Diseases, 6 Sosnovy Blvd, Kemerovo, 650002, Russia; Senior Researcher, Department of Cardiac and Vascular Surgery; Research Institute for Complex Issues of Cardiovascular Diseases, 6 Sosnovy Blvd, Kemerovo, 650002, Russia; Researcher, Department of Cardiac and Vascular Surgery; Research Institute for Complex Issues of Cardiovascular Diseases, 6 Sosnovy Blvd, Kemerovo, 650002, Russia; Junior Researcher, Department of Experimental Medicine; Research Institute for Complex Issues of Cardiovascular Diseases, 6 Sosnovy Blvd, Kemerovo, 650002, Russia; Senior Lecturer, Department of Biomedical Technical Systems, Bauman Moscow State Technical University (National Research University), 5/1 Baumanskaya 2-ya St., Moscow, 105005, Russia; Head of Department, Department of Cardiac and Vascular Surgery, Research Institute for Complex Issues of Cardiovascular Diseases, 6 Sosnovy Blvd, Kemerovo, 650002, Russia

**Keywords:** transcatheter valve replacement, aortic valve, the delivery system of TAVR bioprosthesis, finite element method

## Abstract

**The aim of the study** was to determine the potential mechanism of vascular complications due to “catheter–vascular wall” interaction in transcatheter aortic valve replacement using experimental and numerical analysis.

**Materials and Methods:**

A series of full-scale bench tests and numerical simulations were carried out using the CoreValve commercial transfemoral delivery system for aortic valve bioprosthesis (Medtronic Inc., USA). Full-scale tests were carried out using a phantom of the vascular system (a polymeric silicone model of Transcatheter Aortic Valve; Trandomed 3D Inc., China) with simulation of all stages of delivery system movement along the vascular bed. They involved introduction into the common femoral artery, movement along the abdominal and thoracic parts of the aorta, the aortic arch, and positioning the system to the implantation site. The force arising from the passage of the delivery system was assessed using sensors of a Z50 universal testing machine (Zwick/Roell, Germany). Numerical simulation of transcatheter valve replacement procedure was carried out in a similar way with allowance for the patient-specific anatomy of the recipient’s aorta using the finite element method in the Abaqus/CAE environment (Dassault Systèmes, France).

**Results:**

It was found that in the process of the delivery system passing through the vascular system, there occurred force fluctuations associated with catheter bending and its interaction with the aortic wall in the region of its arch. For example, in the initial straight portions, the pushing force was 3.8–7.9 N; the force increased to the maximum (11.1 and 14.4 N with and without the prosthesis) with bending of the distal portion of the catheter. A similar increase was observed when performing numerical simulation with high-quality graphic visualization of stress on the “spots” of contact between the catheter and the vascular wall with an increase in stress to 0.8 MPa.

**Conclusion:**

Numerical and full-scale bench tests prove the significant effect of the properties of delivery system catheter for transcatheter aortic valve replacement on the interaction with the aortic walls.

## Introduction

Transcatheter aortic valve replacement (TAVR) is now a less invasive alternative option to open surgery for patients with aortic stenosis. The results of large multicenter studies have allowed expanding the indications for TAVR in intermediate-risk patients [1], while improved designs of bioprostheses have made them safer and more convenient. Improvement of TAVR-prostheses made it largely possible to minimize the risks of using innovative valves in comparison with the first generations of such medical devices: paraprosthetic regurgitation is observed nearly 2 times less often [2], conduction disturbances — up to 3 times less often [3]. Nevertheless, some authors believe [4–6] that improving the second TAVR component, the delivery system, will be the key to improving clinical results since vascular injury is considered one of the most common and critical complications for the entire procedure today. This is especially relevant for patients with vascular pathologies — atherosclerotic or calcified lesions of the aortic wall, which might result in damage to the intima, aneurysm, vascular dissection, and rupture in case of overly aggressive interaction with the catheter [7, 8].

Existing approaches to improving delivery systems are largely based on reducing the geometric dimensions — the diameter of the catheter distal portion actually carrying the TAVR prosthesis. This has been confirmed by the evolution of the two most common systems: Edwards Lifesciences, USA (Sapien–Sapien XT–Sapien 3), and Medtronic Inc., USA (CoreValve– Evolut R–Evolut Pro), in which the diameter has been reduced by 1.7 times: from 24 to 14 Fr and from 18 to 14 Fr, respectively [9, 10].

However, stiffness and stress-strain state arising from the interaction between the elements of the biotechnical system of “catheter–vascular wall” are equally important properties that determine the risk of developing vascular complications, especially those leading to fatal outcomes [4].

**The aim of the study** was to determine the potential mechanism of vascular complications in transcatheter aortic valve replacement based on the analysis of interaction within the biotechnical system of “catheter–vascular wall”.

## Materials and Methods

### Full-scale test

**Figure 1:**
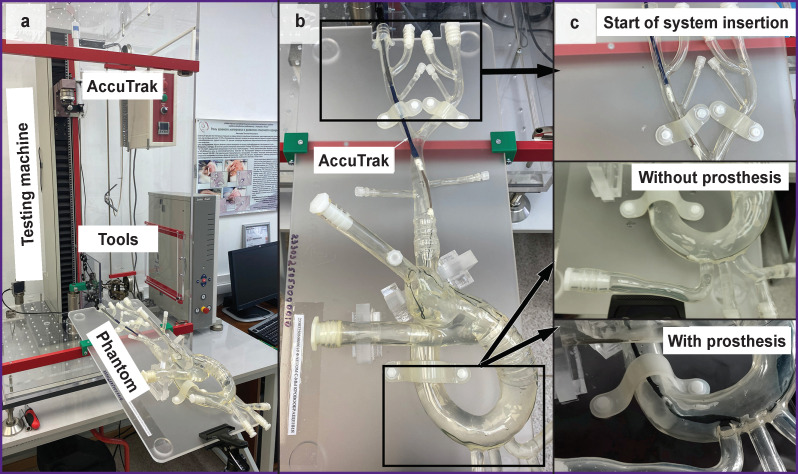
Full-scale test of the AccuTrak transcatheter system (Medtronic Inc.) when simulating the implantation procedure: (a) general setup of the experiment and installation of the system under study in the experimental facility; (b) top view of the vascular system phantom with the initial stage of delivery system advancement; (c) enlarged views of the delivery system in two versions (with and without a packed bioprosthesis)

The object of the study was a CoreValve — AccuTrak transfemoral bioprosthesis delivery system (Medtronic Inc., USA), whose properties were analyzed in the setting of a simulated TAVR procedure using a phantom of the cardiovascular system (a silicone model of Transcatheter Aortic Valve; Trandomed 3D Inc., China). The entire procedure was performed on a Z50 universal testing machine (Zwick/Roell, Germany) (Figure 1 (a)). Investigation of forces arising during movement was carried out when simulating the main stages of catheter insertion by the transfemoral method. These involved introduction into the common femoral artery, movement along the abdominal and thoracic parts of the aorta, the aortic arch, and positioning the system at the implantation site, the aortic valve in the region of the left ventricular outflow tract, which was followed by the controlled extraction of the bioprosthesis (see Figure 1 (a); Figure 1 (b)). A rigid Lunderquist Extra-Stiff wire guide with a diameter of 0.035 inch and a length of 260 cm (COOK MEDICAL LLC, USA) was used in the study.

**Figure 2:**
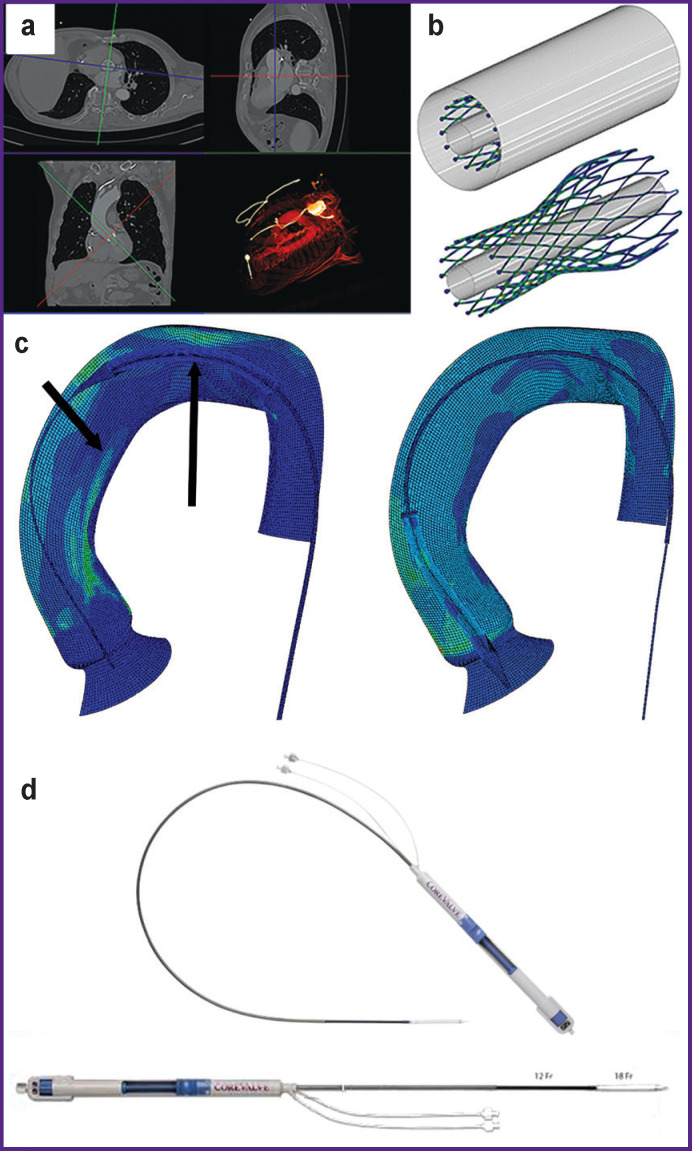
Numerical simulation of transcatheter delivery system advancement: (a) identifying patient-specific anatomy of the aortic root based on clinical data; (b) the stage of crimping the transcatheter prosthesis using an auxiliary surface; (c) stages of delivery system advancement along the guidewire inside the aorta with visualization of contact “spots”; (d) commercial model of the AccuTrak delivery system (Medtronic Inc.)

To ensure conditions close to those natural, the phantom was filled with two liters of liquid — a blood analogue solution (50.3% glycerol; 48.8% distillate; 0.9% NaCl by weight) [11]. To assess the contribution of the prosthesis stiffness to the properties of the delivery system and its “deliverability”, two comparative tests were carried out with and without the prosthesis packed in a delivery sheath (Figure 2 (c)). The general movement of the catheter was provided by changing the position of the traverse of the testing machine while estimating the force required for movement and monitoring the “force–movement” relationship.

### Numerical simulation. Contact

Simulation of the main properties of complex mechanical behavior in the setting of simulated anatomy of the patient’s arteries was carried out on a clinical case of transcatheter aortic valve replacement with a CoreValve bioprosthesis (Medtronic Inc.) performed at the Research Institute for Complex Issues of Cardiovascular Diseases (Kemerovo, Russia) in 2018. The study used de-identified data of multi-slice computed tomography (MSCT) visualizing the anatomy of the aortic root and left ventricle in the period before and after TAVR.

Images (Figure 2 (a)) were obtained using a SOMATOM Sensation 64 MSCT device (Siemens, Germany) under contrast conditions with craniocaudal scan direction, a resolution of 0.625 mm, and ECG synchronization. Three-dimensional personalized models and corresponding meshes of the finite elements (aortic root, including the ascending and descending parts, CoreValve heart valve bioprosthesis, three-dimensional models of the stiff wire guide and delivery system) were obtained in the 3D Slicer environment (USA).

Numerical simulation of the TAVR procedure was carried out in the Abaqus/CAE engineering analysis environment (Dassault Systèmes, France) in three sequential stages:

- packing (crimping) of the CoreValve prosthesis support frame into the delivery system. The final compression by radial movement of the surface was carried out to a diameter of 6.0 mm (18 Fr) (Figure 2 (b));
- advancement of the delivery system to the target site of implantation (see Figure 2 (c));
- release of the support frame in the area of the aortic valve (see Figure 2 (c)).

Friction coefficients were chosen according to the literature: 0.0384 for the “catheter–vessel” pair [12] and 0.035 for the “guide–catheter” pair [13]. The qualitative criterion for verification was recording of intraoperative aortography, visualizing the stages of the delivery — movement along the aortic arch and root.

### Numerical simulation. Stiffness

The study of correlation between the stiffness of the distal portion of the delivery system, the pushing force, and the stress-strain state of the aortic wall was carried out numerically, setting five variations in the stiffness of the distal portion of the catheter system. The initial reference stiffness value was that resulting from the full-scale experiment, defining this parameter as the inverse of the force applied during catheter advancement. The physical and mechanical properties of the distal portion of the catheter model were changed during numerical simulation in such a way as to obtain the following variations in stiffness — 0.25, 0.5, 2.0, 4.0, 8.0 from the original. Simulation was performed for the patient-specific case described above, analyzing similar parameters: contact pressure in “catheter–vascular wall” pair, stress-strain state of the components (prosthesis, aortic root, delivery system catheter).

## Results

### Full-scale test

**Figure 3:**
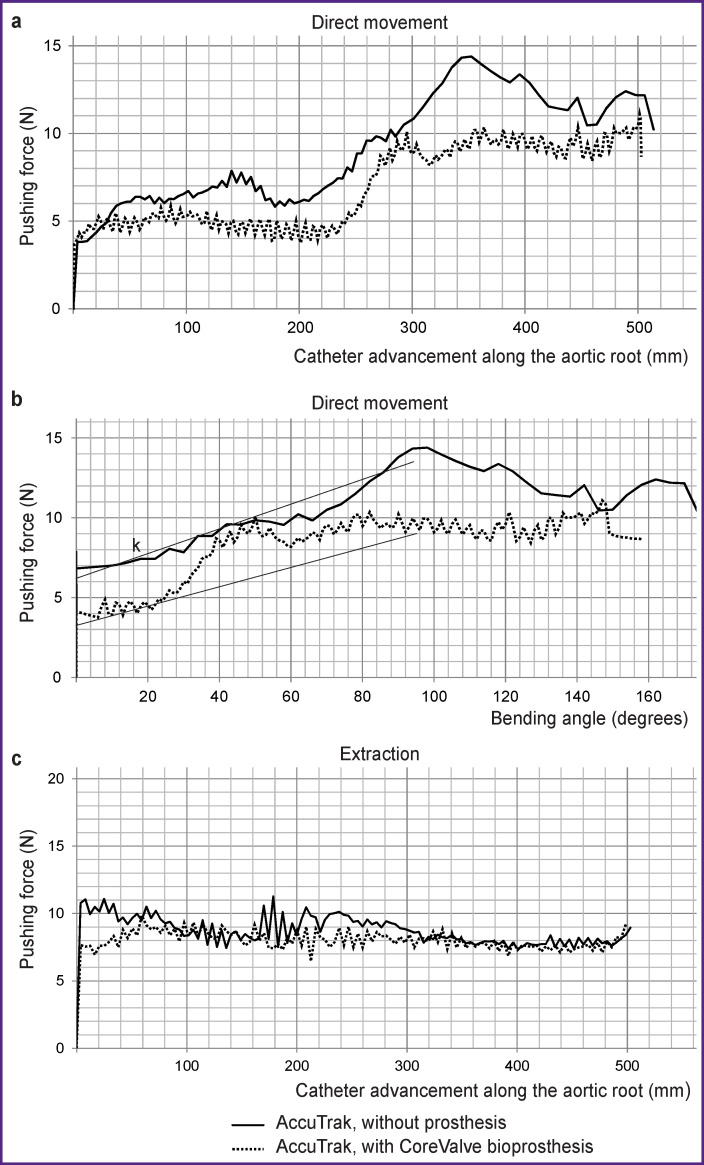
Quantitative results of a full-scale test of the AccuTrak transcatheter system (Medtronic Inc.) during simulation of implantation procedure using a polymer phantom on a Z50 universal testing machine (Zwick/Roell) for two cases — with and without a crimped prosthesis: (a) “pushing force–catheter advancement” relationship in the forward direction; (b) “pushing force–catheter bending angle” dependence in the forward direction (k — visualization of delivery system stiffness coefficient); (c) quantitative data of “force–displacement” testing during transcatheter delivery system extraction

**Figure 4:**
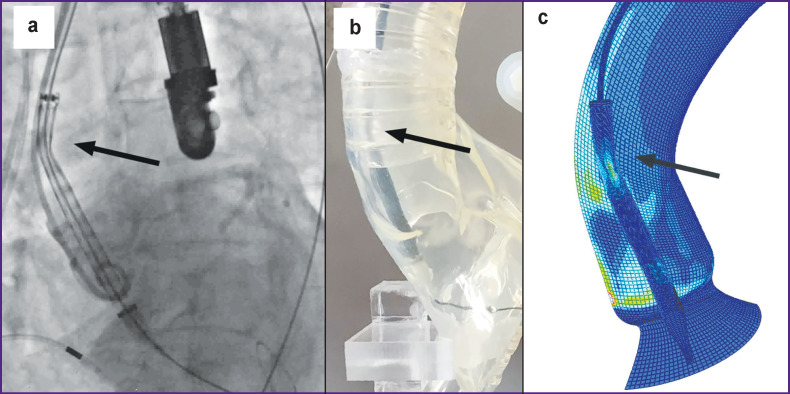
Qualitative visualization of features of transcatheter system under study: (a) intraoperative aortography during the TAVR procedure; (b) enlarged photo during a full-scale test on a phantom of the circulatory system; (c) visualization of the intermediate stage of introducing the delivery system into the aortic root in patient-specific TAVR modeling. The arrows indicate zones of bending taking place in the distal part of the delivery system

As a result of the study, it was found that the force created by the catheter during movement was 3.9–14.4 N, depending on the zone of the vascular phantom passed by the delivery system, with a gradual expected increase in the friction force as the contact areas of “catheter–phantom wall” and “catheter–guide” expanded. When passing through the aortic arch, the maximum effort in both cases (with and without the prosthesis) was 11.1 and 14.4 N, respectively (Figure 3 (a)). The main increase in forces was determined by the increase in delivery system deformation due to bending of the catheter in the region of the aortic root: the graph “force–catheter bending angle” clearly demonstrates the increase in forces with a spike of more than 30% when a bend of more than 20° occurs (Figure 3 (b)). Notably, bending of the proximal quarter of the delivery sheath characteristic of this delivery system was well observed, the bending being more significant for the case without a packed prosthesis (Figure 4 (a)).

The reverse movement of the delivery system, its extraction, also demonstrated inhomogeneity of force changes depending on the vascular bed portion, however, there was no expected significant drop in forces observed after the catheter passed through the aortic arch. Thus, the onset of extraction was characterized by a force range of 7–9 N, a portion of the aortic arch — by an increase in force to 10.0–10.5 N, and the final straight portion — by a return to the range of 7.0–9.0 N (Figure 3 (c)). Besides, the force ranges did not differ significantly in the two study options — with and without a heart valve prosthesis packed into the delivery system, as well as in the area of the aortic arch where direct movement (implantation) showed significant differences. Significant differences (fluctuations) of forces up to 2.40–3.15 N were recorded all through the extraction stage.

It should be noted that an important limitation of a full-scale bench experiment to determine the pushing force was the properties of the “vascular” wall of the phantom (silicone), which differ from the properties of the native aorta mainly in the friction coefficient. The use of a liquid (an analogue of blood) in the experiment reduced the “silicone–catheter” friction coefficient, however, even in this case, when the delivery system moves along the phantom, the value of the force amplitude may differ from the experiment with native tissues and actual implantation. We hypothesized that numerical simulation using properties similar to those of the aorta, including the more physiological “catheter–aorta” friction coefficient of 0.0384 [12], was a more valid source of quantitative force values.

### Numerical simulation. Contact.

**Figure 5:**
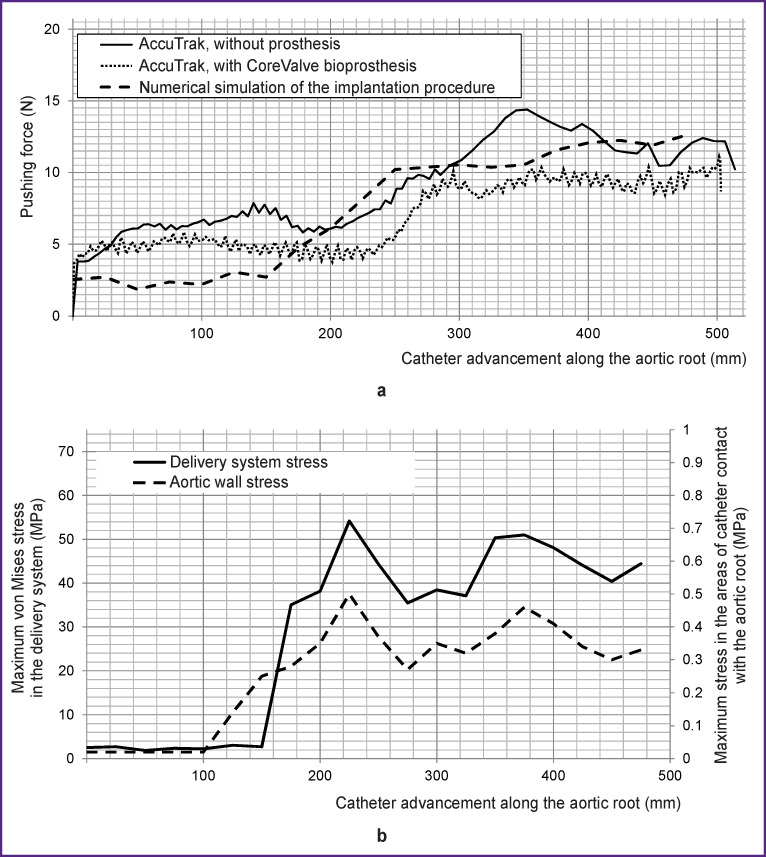
Quantitative results of numerical simulation of a patient-specific procedure for transcatheter aortic valve replacement: (a) evaluation of the pushing force obtained as a result of simulation in comparison with the results of the full-scale test with a phantom of the circulatory system (two cases); (b) maximum von Mises stress in the delivery system and the aortic wall when moving the catheter along the vascular bed

It was found during simulation, that when the delivery system moved inside the catheter, there was an insignificant change in the stress-strain state of the support frame, unable to exceed the deformation occurring on crimping even for bends of the proximal portion (in the region of the aortic root). For example, the deformation values at the stage of packing (crimping) amounted to 3.4–6.1%, while the increase in this parameter at the stage of forwarding the delivery system to the target site of implantation was 0.4–0.7%. Analysis of the stress-strain state of the tubular delivery components, including that in the distal bending elements, revealed no significant critical values exceeding the threshold: the maximum von Mises stress was 54.1–91.8 MPa, depending on the region of the delivery system, with the lowest threshold rupture values obtained during uniaxial testing equaling 216.1–315.1 MPa (Figure 5 (a)).

According to the simulation results, the applied catheter movement forces are in qualitative agreement with the results of full-scale tests using a phantom of the cardiovascular system (see Figure 5 (a)). The tendency to change the forces is also associated with catheter bending in the aortic root: the increase in forces reached 150% as compared to the straight portions of the vascular system. However, in terms of quantity, the amplitudes of forces obtained during computer simulation were significantly smaller than for the phantom, which was attributed to a different, more physiological friction coefficient in the calculations.

### Numerical simulation. Stiffness.

**Figure 6:**
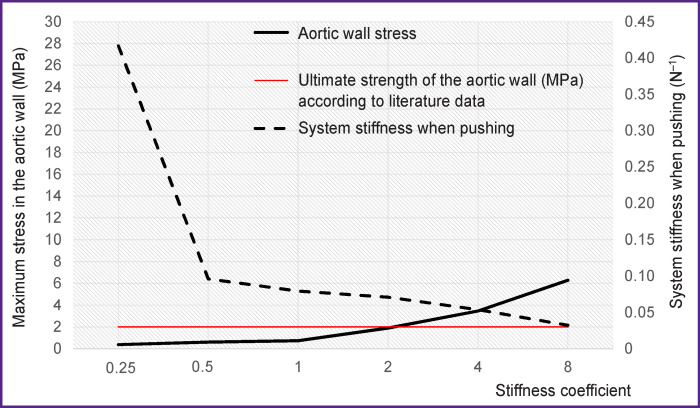
Quantitative relationship between the stiffness of the distal region of the AccuTrak delivery system (Medtronic Inc.), stress-strain state of the aorta, and the pushing force of the catheter in numerical simulation

Nonlinear relationships of “stiffness–maximum pushing force” and “stiffness–stress of the aortic root” were obtained on simulation (Figure 6). It was revealed that an artificial increase in the catheter stiffness by 4 and 8 times significantly increased the maximum stress arising in the wall of the aortic arch in the contact areas by 4.7 (up to 3.47 MPa) and 8.5 (up to 6.28 MPa) times. This growth can be associated with a significant increase in the likelihood of vessel wall injury. On the other hand, the increase in stiffness did not cause such a strong increase in the pushing force of the catheter (an increase of 1.5 and 2.4 times, respectively), i.e. it did not significantly affect the catheter’s ability to move along the aortic root. Reducing the catheter stiffness by 2 times reduced aortic wall stress by 17.4–49.0%, also having a small effect on the pushing force (decrease by 17.5–73.9%).

## Discussion

Simulation of transcatheter valve replacement procedure using a silicone phantom model with a commercial delivery system made it possible to quantify the forces arising during implantation and to describe the dynamics of their change during catheter movement along the vascular bed. It was found that straight portions of the vascular system create nonzero (3.9–5.0 N) resistance to catheter advancement due to interaction with the underlying areas — the access site and contact in the area of the aortic bifurcation, as well as the presence of friction in “catheter–guide” contact pair. As the catheter moved further, an additional contribution to the formation of the forward force was made by catheter deformation component and an increase in “catheter–vascular wall” friction force. As a result, the forces required to forward the delivery system reached 11.1–14.4 N in the area of the aortic arch with a slight decrease when passing through this area.

It was found in the numerical experiment that during the movement along the vascular bed, there was a direct contact between the delivery system and the aortic wall, which is undesirable since it can create stress concentrators (“spots”) in the aorta. These areas of potential vascular complications are localized in two key areas: the “descending aorta–aortic arch” junction and the area of the sinotubular junction. The emergence of contact “spots” with a voltage of up to 0.8 MPa was observed in these areas passed by the delivery catheter (Figure 4 (b)). This effect is likely associated with the passage of the guidewire close to the anatomical structures due to bending.

The second part of numerical simulation — an artificial change in catheter system stiffness — clearly demonstrated the relationship between catheter properties and the characteristics of “catheter–aorta” contact interaction, the amplitude of stress in that “spot”. In some cases, the stress of the aortic wall increased above its ultimate strength (~2 MPa [14]) by 73.5–325.0% (up to 3.47–8.50 MPa) with an increase in catheter system stiffness, which clearly evidenced the risk of vessel injury. This mechanism may explain the emergence of injury-associated (more exactly, dissection-associated) complications of the ascending and descending regions of the aortic root in clinical practice, especially in the presence of atherosclerotic or calcified changes potentially reducing the ultimate strength of the vessel wall. However, it remains unclear what causes complications in the underlying areas, for example, the abdominal area, for which no significant “catheter–vessel wall” interaction was revealed in this study, while there is clinical evidence of injury [4].

In general, the quantitative data obtained are similar to those from analogous studies devoted to testing transcatheter systems. The study of 15 cadaveric ascending aortas performed by Heinisch et al. [8] showed that forces arising during the transfemoral approach also significantly varied depending on the part of the vascular bed (see the Table). Nevertheless, force values obtained in the present study are slightly higher than in the work compared, especially for the case of a delivery system without a packed prosthesis. This difference is attributable to different experimental models: comparison of forces was carried out using a natural organ (aortic root) in the case of Heinisch et al., while a silicone polymer phantom was used in this work. These objects differ in properties, primarily in friction coefficient, although the differences do not seem to be dramatic.

**Table:** Catheter pushing force (N) (Me [min–max])

Segment	Heinisch et al. [8]	The authors’ own data (full-scale bench tests)
From the iliac artery to aortic bifurcation:		
without prosthesis	3.6 (1.0–6.0)	6.4 (3.8–7.9)
with prosthesis	3.0 (1.8–4.6)	5.0 (3.7–5.9)
From the abdominal aorta to the left subclavian artery:		
without prosthesis	4.9 (2.0–10.0)	7.1 (5.8–10.5)
with prosthesis	5.6 (3.4–7.6)	4.9 (3.8–10.1)
From the aortic arch to the ascending aorta:		
without prosthesis	12.1 (7.0–17.0)	12.2 (10.2–14.4)
with prosthesis	11.5 (6.7–17.0)	9.3 (8.2–11.1)

## Conclusion

The results obtained demonstrate a significant effect of the properties of TAVR delivery system catheter on the interaction with the aortic walls according to numerical and full-scale tests. Increasing the stiffness of delivery system components can cause a significant increase in the pushing force of the catheter, i.e. an increase in the force of friction, and lead to the occurrence of contact “spots” with high stress amplitude. Notably, the most critical area where these effects take place is the aortic arch as the catheter passes close to the vessel wall and their contact is likely to occur. These facts suggest that the mechanism of damage to the inner wall of the aorta is activated at significant stress amplitudes in this area, which can be reduced by lowering catheter stiffness.
